# Comparative genomic analysis of *Fusobacterium nucleatum* reveals high intra-species diversity and cgmlst marker construction

**DOI:** 10.1186/s13099-023-00570-z

**Published:** 2023-09-14

**Authors:** Qianhui Zhu, Arslan Dovletgeldiyev, Chen Shen, Kexin Li, Songnian Hu, Zilong He

**Affiliations:** 1 School of Engineering Medicine, Beihang University, No. 37 Xueyuan Road, Haidian District, Beijing, 100191 China; 2 Beijing Advanced Innovation Center for Big Data-Based Precision Medicine, Interdisciplinary Innovation Institute of Medicine and Engineering, Beihang University, Beijing, China; 3 State Key Laboratory of Microbial Resources, Institute of Microbiology, Chinese Academy of Sciences, Beijing, China; 4 Departmant of Urology, The Second Affiliated Hospital of Dalian Medical University, Dalian, 116023 Liaoning China; 5 Systems Biology and Bioinformatics (SBI), Leibniz Institute for Natural Product Research and Infection Biology—Hans Knöll Institute (HKI), Jena, Germany

## Abstract

**Background:**

*Fusobacterium nucleatum* is a one of the most important anaerobic opportunistic pathogens in the oral and intestinal tracts of human and animals. It can cause various diseases such as infections, Lemierre's syndrome, oral cancer and colorectal cancer. The comparative genomic studies on the population genome level, have not been reported.

**Results:**

We analyzed all publicly available *Fusobacterium nucleatum*s’ genomic data for a comparative genomic study, focusing on the pan-genomic features, virulence genes, plasmid genomes and developed cgmlst molecular markers. We found the pan-genome shows a clear open tendency and most of plasmids in *Fusobacterium nucleatum* are mainly transmitted intraspecifically.

**Conclusions:**

Our comparative analysis of *Fusobacterium nucleatum* systematically revealed the open pan-genomic features and phylogenetic tree based on cgmlst molecular markers. What’s more, we also identified common plasmid typing among genomes. We hope that our study will provide a theoretical basis for subsequent functional studies.

**Supplementary Information:**

The online version contains supplementary material available at 10.1186/s13099-023-00570-z.

## Introduction

*Fusobacterium nucleatum* is a Gram-negative bacterium, one of the most important anaerobic opportunistic pathogens in human and animals, and is mainly found in the oral and intestinal tracts [1]. *Fusobacterium nucleatum* can cause periodontal disease, acute necrotizing gingivitis, oral cancer, ulcerative colitis, crohn's disease and colorectal cancer, and even changes in the local inflammatory environment [2]. *Fusobacterium nucleatum* can lead to overgrowth of non-functional tissues, hence the name "oncobacterium" [3]. *Fusobacterium nucleatum* is highly toxic as it produces lipopolysaccharides (LPS), endotoxins and haemolysins [4]. Although it is part of the normal microbiota of human tissues, it can invade tissues following surgical or accidental trauma, oedema, hypoxia and/or tissue destruction and is highly pathogenic [5].

The biological functions of *Fusobacterium nucleatum* are currently being studied in depth. It is one of the few non-spore-producing anaerobic species that uses amino acid catabolism to provide energy, using glutamate, histidine and aspartate [6, 7]. Its metabolism naturally increases the pH of its local environment by consuming amino acids and releasing ammonia, thereby enabling the growth of acid-sensitive bacteria such as *Porphyromonas gingivalis* [8]. *Fusobacterium nucleatum* has an outer membrane with a large number of proteins on its outer cell surface, and specific interactions can be found between the bacteria and various complementary structures on the surface of the host cell [9]. This adhesion is mediated by adhesion factors. This adherence is important for the colonisation and establishment of infection in susceptible hosts. Adhesion A (FadA) is a bacterial hair adhesion protein that has recently been shown to be required for bacterial attachment and invasion of the gingival epithelium and endothelium [10]. It is conserved in the genus *Fusobacterium* that inhabits the oral mucosa and is important for cell binding [11]. It has been demonstrated that *Fusobacterium nucleatum* is an important contributor to oral biofilm development [11]. In addition to oral diseases, *Fusobacterium nucleatum* has been reported to be associated with a variety of intestinal diseases [3]. A meta-analysis showed that *Fusobacterium nucleatum*’s DNA was more likely to be detected in colorectal tumour tissue compared to adjacent healthy tissue and control tissue [12]. Its DNA was also higher in colorectal polyp tissue compared to healthy tissue in the control group [12]. In another study, *Fusobacterium nucleatum* was shown to mediate the development of colon cancer and the concomitant metastasis of the tumour [13]. In summary, the studies of *Fusobacterium nucleatum* have focused on biological mechanisms, but comparative genomic studies in this species, particularly the population genome level, have not been reported. Meanwhile, due to pubmlst database does not contain mlst gene markers for *Fusobacterium nucleatum*, so the development of cgmlst molecular markers with high resolution for this species is required.

In this study, we collected all publicly available *Fusobacterium nucleatum*s’ genomic data for a comparative genomic study, focusing on the pan-genomic features, virulence genes and plasmid genomes of the species and developed cgmlst molecular markers for the species, with the aim of providing a theoretical basis for subsequent identification and in-depth functional studies of *Fusobacterium nucleatum*.

## Materials and methods

### Public data acquisition and quality control

The *Fusobacterium nucleatums*’ genomic data included in this study for genomic analysis were all downloaded from the NCBI database (https://ftp.ncbi.nlm.nih.gov/genomes/genbank/bacteria/Fusobacterium_nucleatum/). Phenotype information was also obtained from the NCBI database. The genomic data were downloaded and evaluated for quality of assembly and core gene content using QUAST (version 5.2.0) [14] and BUSCO (version 5.4.3) [15] software, respectively, with > 90% integrity, < 5% contamination and < 500 scaffolds. The above software used default parameters in the analysis.

### Genome annotation and pan-genome construction

The filtered genomes were used to construct a pangenome of *Fusobacterium nucleatum*. The genomes were firstly annotated using Prokka (version 1.14.6) [16] and the *Fusobacterium nucleatum* pangenome was constructed using Roary (version 3.10.2) [17] based on the genome annotation file (gff3 file). We classified core/ unique genes by using Roary with default parameters (95% identity for blastp and 99% of isolates a gene must be in to be core). We drew pan genome plot of *Fusobacterium nucleatum* by using Pan-GP [18]. Both Prokka and Roary used default parameters in the analysis. The core gene set and cgmlst molecular marker construction were based on the gene_presence_absence.csv file from Roary's results. Functional annotation of the core gene set was enriched using KAAS [19] (https://www.genome.jp/tools/kaas/). The phylogenetic tree was constructed by using the ‘core_gene_alignment.aln’ from Roary result. We used the Fasttree to generate the phylogenetic tree with 1000 replications [20]. The evolutionary tree of the genomes was visualized using iTOL [21].

### Key genes prediction and evolutionary tree construction

Virulence genes were predicted by using blastp based on the VFDB database [22] (http://www.mgc.ac.cn/VFs/). Resistance genes were predicted by the CARD database [23] (http://arpcard.mcmaster.ca). The parameters of blastp were e value 1e-5, similarity 60%, qcov 60% and tcov 60%. The FadA gene protein sequence was extracted based on the annotation results from Prokka. Multiple sequence alignment of the FadA protein sequence was performed using mafft, evolutionary tree construction was performed by MEGA [24] and evolutionary tree annotation was performed using iTOL.

### Plasmid genome prediction and genomic analysis

Plasmer software (https://github.com/nekokoe/plasmer) and Platon were used to perform plasmid prediction on the whole genome sequence of *Fusobacterium nucleatum* after quality control [25]. The predicted sequences were verified by blast against a non-redundant nucleic acid library from NCBI. The circular represent map of the plasmids was visualized using CGview [26].

## Results

### Summary of genomic data

A total of 105 *Fusobacterium nucleatum*s’ genome sequences were downloaded from the NCBI, quality controlled and evaluated for core genes. Finally, 93 genomes were selected for further analysis (Additional file 5: Table S1). The number of scaffolds ranged from 1 to 379, with a maximum N50 value of 2,653,055 bp, a minimum value of 8,680 bp and an average genome size of 2,369,555 bp. We collected the metadata of the downloaded strains (Additional file 6: Table S2) and found that *Fusobacterium nucleatum* was isolated mainly from the oral cavity (N = 33) and the intestine (N = 16), excluding the majority of strains with unknown phenotypes.

### Pan-genomic characterization of *Fusobacterium nucleatum*

**Fig. 1:**
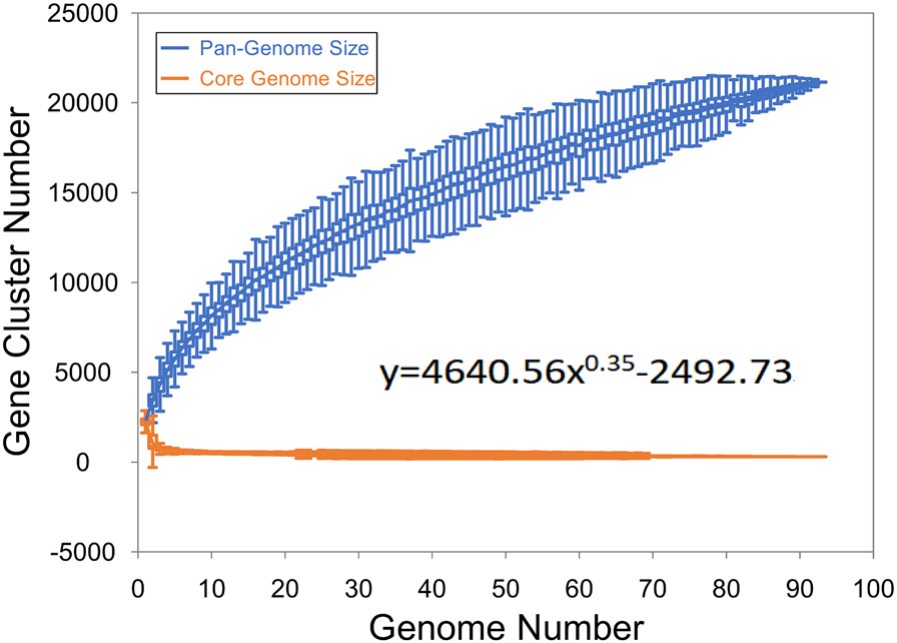
The pan genome plot of *Fusobacterium nucleatum.* **A**. Conserved genes and Total genes. **B**. New genes and Unique genes

A total of 93 *Fusobacterium nucleatum* strains were included in the first pangenome analysis. The *Fusobacterium nucleatum* genome contains a total of 21,139 gene families, of which 516 are core (present in more than 95% of the genome). The number of variable gene families is 20,623. According to Fig. 1, the pan-genome shows a clear open tendency, and the size of the pan-genome continues to increase with the number of genomes included in the analysis, showing a continuous upward trend. The number of emerging gene families in the pan-genome increases with the number of genomes, and in turn the size of the pan-genome will expand. The heatmap of gene presence-absence matrix showed two distinct clades in *Fusobacterium nucleatum* (Additional file 1: Figure S1)*.*

Based on the core gene set, we constructed a cgmlst molecular marker (N = 298) for *Fusobacterium nucleatum* (Additional file 7: Table S3A) and a phylogenetic tree for 93 strains based on this markers (Additional file 2: Figure S2). The phylogenetic tree showed that there were no obvious clades of *Fusobacterium nucleatum* and, based on the known meta information, the strains from the oral cavity as well as the intestine were scattered and did not aggregate significantly. Functional enrichment of these 298 genes showed that they were mainly derived from the Ribosome and ABC transporters pathways (Additional file 3: Figure S3). Notably, we also attempted to construct separate cgmlst molecular markers from the oral cavity and intestine (Additional file 7: Table S3BC), and the Venn diagram shows that these two types of markers share 384 genes, while the oral cavity (N = 16) and intestine (N = 161) each retain a small number of cgmlst genes (Additional file 4: Figure S4).

### Bioinformatic analysis of virulence genes and FadA gene

**Fig. 2:**
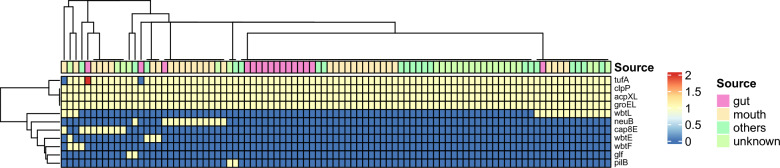
Heatmap of virulence related genes in *Fusobacterium nucleatum*

We examined the virulence genes in the genomic data of 93 *Fusobacterium nucleatum* strains based on the VFDB database (Fig. 2). A total of 11 virulence genes were found to be present in the genome, notably groEL, clpP and acpXL were found to be present in 93 strains with copy number 1. tufA was present in most strains, while other virulence factors such as cap8E, neuB and wbtE were present in a small number of strains. In addition, we also predicted drug-resistant genes for these strains and found that the majority of *Fusobacterium nucleatum* did not carry those genes, but were present in only a few strains.

**Fig. 3:**
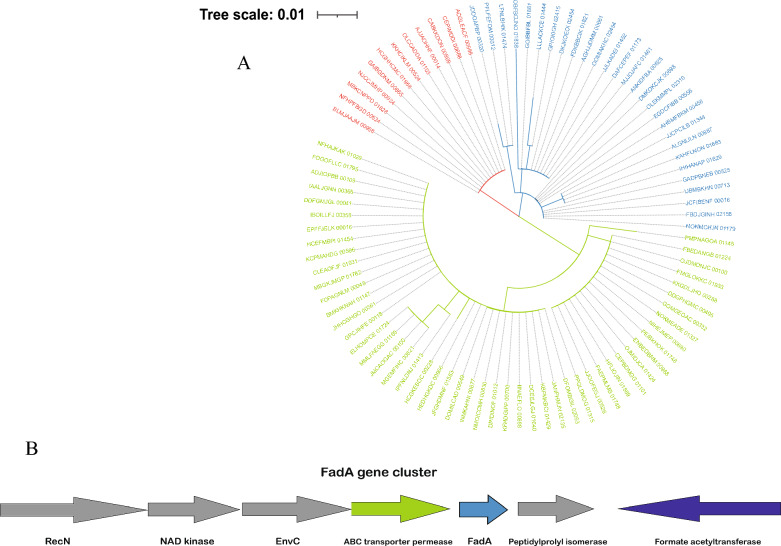
Genomic analysis of FadA genes in *Fusobacterium nucleatum.* **A**. The phylogenetic tree of FadA genes. **B**. the genomic structure of FadA genes

We also analyzed the *Fusobacterium nucleatum* genomes for the FadA genes, a hair adhesion protein that is important for cell binding. We found that 90 of these strains contained the FadA gene in their genome sequences and, based on the FadA protein sequence, we constructed a phylogenetic tree that showed three distinct clades of the FadA gene, with strains from the oral and intestinal tracts in each of the three clades (Fig. 3A). In addition, we investigated the upstream and downstream structure of the FadA gene and found that the upstream and downstream structure of the FadA gene is relatively conserved in *Fusobacterium nucleatum* genomes, with the FadA gene surrounded by ABC transporter permease and Peptidylprolyl isomerase, and upstream and downstream genes such as EnvC and NAD kinase (Fig. 3B).

### Plasmid prediction and genomic analysis of *Fusobacterium nucleatum*

**Fig. 4:**
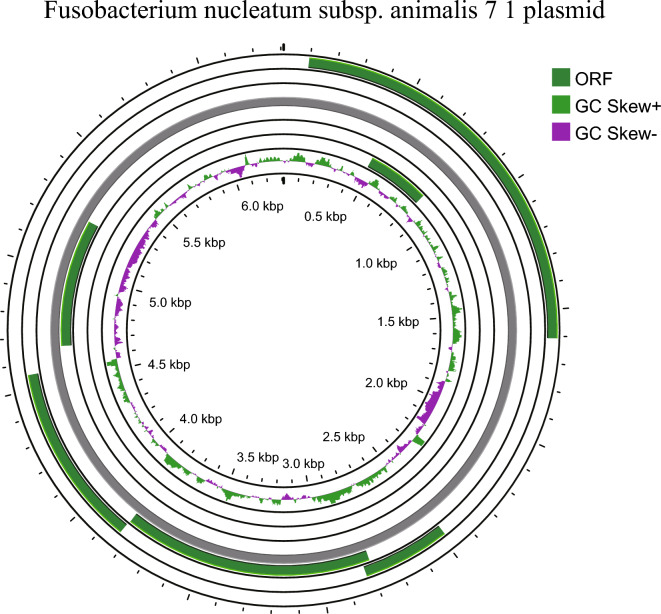
The circular representation map of 7–1 plasmid

We have used the newly developed plasmid prediction tool Plasmer to predict the genome sequences of *Fusobacterium nucleatum*. In total, we found plasmid sequences in the genomes of 42 strains. We then filtered plasmid sequences with high quality genomes for subsequent analysis (number of contigs < 3) and validated the plasmids based on the NCBI non-redundant nucleic acid library. In total, we identified 17 strains with relatively complete plasmid sequences present. Of these plasmid genomes, 13 are known, and in addition we identified four unreported sequences of around 15 K in length, which we speculate are likely to be newly discovered plasmid sequences (Table 1). Among the known plasmid genomes, five strains carry plasmid type 7–1, while other plasmid types include 4–8, pFN3 and pCT15E1. 7–1 plasmid has a genome size of 6.3 K and contains a total of seven mRNA-encoding genes, most of which are putative proteins, with no resistance or virulence genes identified (Fig. 4).

**Table 1:** The predicted plasmids of *Fusobacterium nucleatum*

GCA ID of Fn isolates	Annotation	Genome length
GCA_022340045.1	Clostridioides difficile strain UK 012 plasmid unnamed1	31181
GCA_002591465.1	*Fusobacterium nucleatum* plasmid pKH9	5054
GCA_000400875.1	*Fusobacterium nucleatum* subsp. animalis 4_8	14194
GCA_000158275.2	*Fusobacterium nucleatum* subsp. animalis 7_1	6309
GCA_000234075.2	*Fusobacterium nucleatum* subsp. animalis 7_1	6309
GCA_000273605.1	*Fusobacterium nucleatum* subsp. animalis 7_1	6309
GCA_000273625.1	*Fusobacterium nucleatum* subsp. animalis 7_1	6309
GCA_902373855.1	*Fusobacterium nucleatum* subsp. animalis 7_1	6309
GCA_001457555.1	*Fusobacterium nucleatum* subsp. polymorphum ATCC 10953 plasmid pFN3	11934
GCA_000153625.1	*Fusobacterium nucleatum* subsp. polymorphum ATCC 10953 plasmid pFN3	11934
GCA_001455125.1	*Fusobacterium nucleatum* subsp. polymorphum strain ChDC F319 plasmid unnamed2	5079
GCA_019552045.1	*Fusobacterium nucleatum* subsp. polymorphum strain THCT15E1 plasmid pCT15E1	11001
GCA_000163915.2	*Fusobacterium nucleatum* subsp. vincentii 3_1_27 plasmid	15921
GCA_002573475.1	Potential novel plasmids	13048
GCA_002591475.1	Potential novel plasmids	14379
GCA_002761915.1	Potential novel plasmids	13120
GCA_022340115.1	Potential novel plasmids	15564

## Discussion

Most studies on *Fusobacterium nucleatum* have focused on its biological functions and the genome of individual bacteria, but the population genomes of this species has not been reported. In this study, a pan-genomic characterization of *Fusobacterium nucleatum* was constructed for the first time based on the genomic data of about 100 strains publicly available to provide a panoramic view from a population perspective. From a pan-genomic view, the core gene family of this species was 516, accounting for 23% of the total number of genes per strain on average. The low proportion of core gene families, combined with the results in Fig. 1, show that the total number of genes as well as the number of unique genes in this species did not show a flat trend, suggesting that the genome is very plastic and that the available number of strains may not allow a complete assessment of the overall pan-genomic trend of *Fusobacterium nucleatum*.

The development of a set of molecular markers for *Fusobacterium nucleatum* identification is important as several studies have reported the association of this species with the development of various diseases such as infections, Lemierre's syndrome, oral cancer and colorectal cancer [3]. As the *Fusobacterium nucleatum* genome has not been well studied, no previous studies have designed mlst molecular markers for this species and no ST typing for this strain has been included in the pubmlst database. With the development of sequencing technology, more and more genomic data of *Fusobacterium nucleatum* will be available. In this study, the first attempt was made to construct the cgmlst molecular markers for this species. Compared with traditional mlst markers, cgmlst markers has the advantages of good universality and high resolution. Since the genome size and phenotypic information of *Fusobacterium nucleatum* are currently inadequate, the clades and the corresponding phenotypic association studies need to be strengthened.

In this study, the virulence genes of *Fusobacterium nucleatum* were studied in detail. Three virulence genes, groEL, clpP and acpXL, were found to be present in each strain. groEL was shown to be involved in the adhesion or invasion of various target cells or tissues [27]. clpP is a serine protease involved in proteolysis [28], while acpXL is an acyl carrier protein [29]. They play a role in the adhesion and invasion of *Fusobacterium nucleatum*. In addition, virulence genes such as Elongation factor Tu, Glucose-1-phosphate thymidylyltransferase and Type 8 capsular polysaccharide synthesis protein were also present in some of the strains. *Fusobacterium nucleatum* has previously been reported to produce β-lactamases [30], which were not found in our study, and this may be related to individualized differences in strains and numbers.

In this study, we used the software named Plasmer in github (https://github.com/nekokoe/plasmer) to perform plasmid prediction on *Fusobacterium nucleatum* genomic data. The results showed that 13 of the high-quality plasmid predictions were identical to plasmids in known public databases, indicating the high accuracy of the software. Overall, the plasmid genomes of *Fusobacterium nucleatum* averaged under 20 k, with most plasmids coming from this species and few from other bacteria, which may indicate that plasmid are mainly transmitted intraspecifically. In addition, no resistance or virulence genes were detected in these plasmids.

However, there are some shortcomings in this study. Firstly, only the genomic functions of *Fusobacterium nucleatum* at strain level are explored, without combining metagenomic data to reveal the abundance of this species in the microbial community and its interactions with other species. In addition, the transcriptional expression of key genes of this species, such as the FadA gene, has not been demonstrated. These issues will be elucidated in subsequent studies.

## Conclusion

Our comparative analysis of *Fusobacterium nucleatum* based on publicly available data reveals a distinct open tendency of the pan-genome and identifies cgmlst molecular markers for this species. We systematically analyzed the virulence gene profile and focused on the upstream and downstream structure and evolutionary relationships of the FadA gene. In addition, we predicted the plasmid sequences in *Fusobacterium nucleatum* and identified common plasmid typing among them. In conclusion, we hope that our study will provide a theoretical basis for subsequent functional studies and clinical applications of *Fusobacterium nucleatum*.

### Supplementary Information

**Additional file 1: Figure S1.** The heatmap of gene presence-absence matrix in *Fusobacterium nucleatum*.

**Additional file 2: Figure S2.** The phylogenetic tree of *Fusobacterium nucleatum* based on cgmlst markers. (Red represents mouth isolates, dark green represents gut isolates, blue represents other isolates and green represents unknown isolates.).

**Additional file 3: Figure S3.** Functional enrichment of cgmlst marker genes.

**Additional file 4: Figure S4.** Venn diagram of cgmlst markers from mouth and gut isolates of *Fusobacterium nucleatum*.

**Additional file 5: Table S1.** The basic statistics of *Fusobacterium nucleatum* genomes.

**Additional file 6: Table S2.** Metadata of *Fusobacterium nucleatum* genomes.

**Additional file 7: Table S3.** (A) cgmlst marker of *Fusobacterium nucleatum* genomes. (B) cgmlst marker of *Fusobacterium nucleatum* genomes isolated from mouth. (C) cgmlst marker of *Fusobacterium nucleatum* genomes isolated from gut.

## Data Availability

The data set analyses during the current study are available in the NCBI database.
