# Supplementary figures and images for: Comparative genomic analysis of Fusobacterium nucleatum reveals high intra-species diversity and cgmlst marker construction
Source: Gut Pathog. 2023 Sep 14;15:43. doi: 10.1186/s13099-023-00570-z (PMC10503116; doi:10.1186/s13099-023-00570-z)

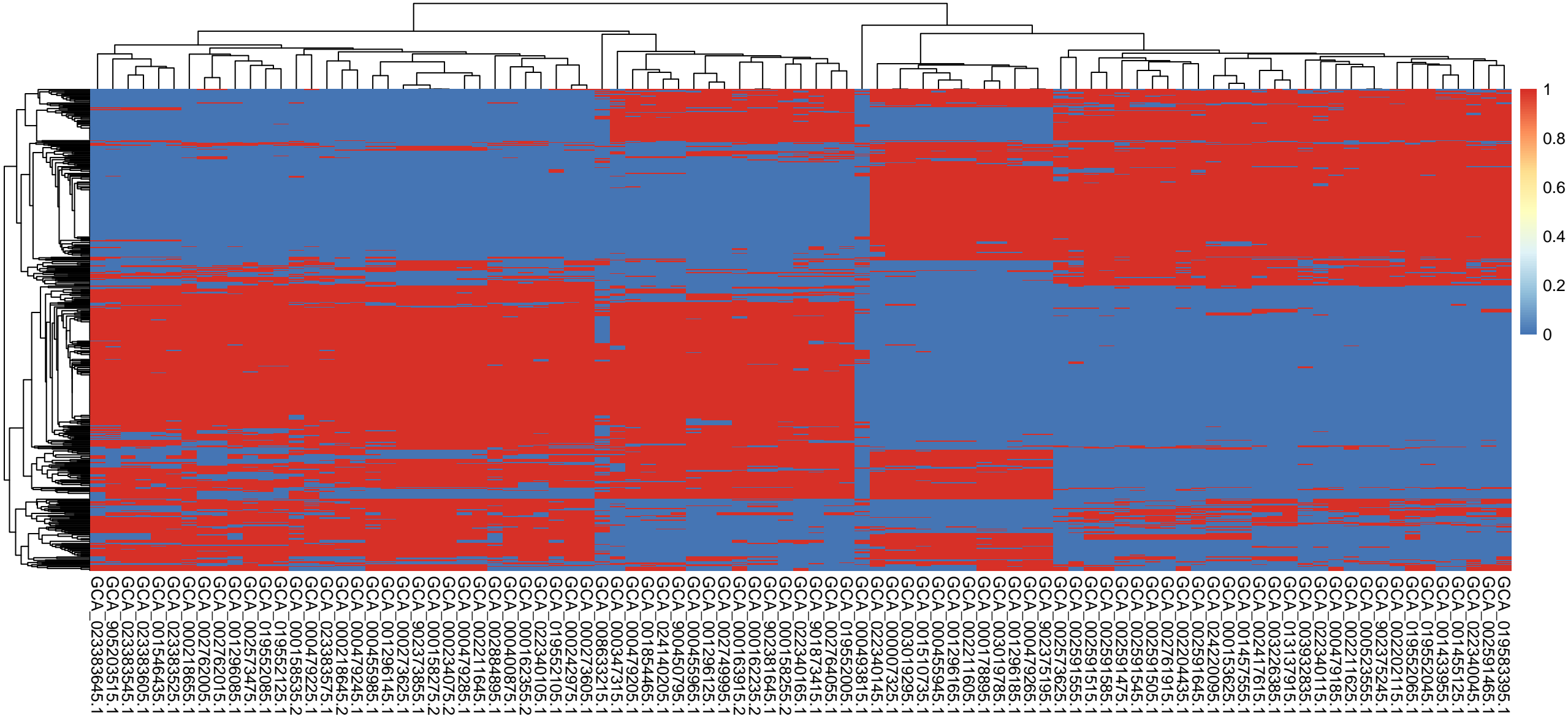

Supplement: Supplementary file 1 — Additional file 1: Figure S1. The heatmap of gene presence-absence matrix in Fusobacterium nucleatum. [file 13099_2023_570_MOESM1_ESM.pdf]

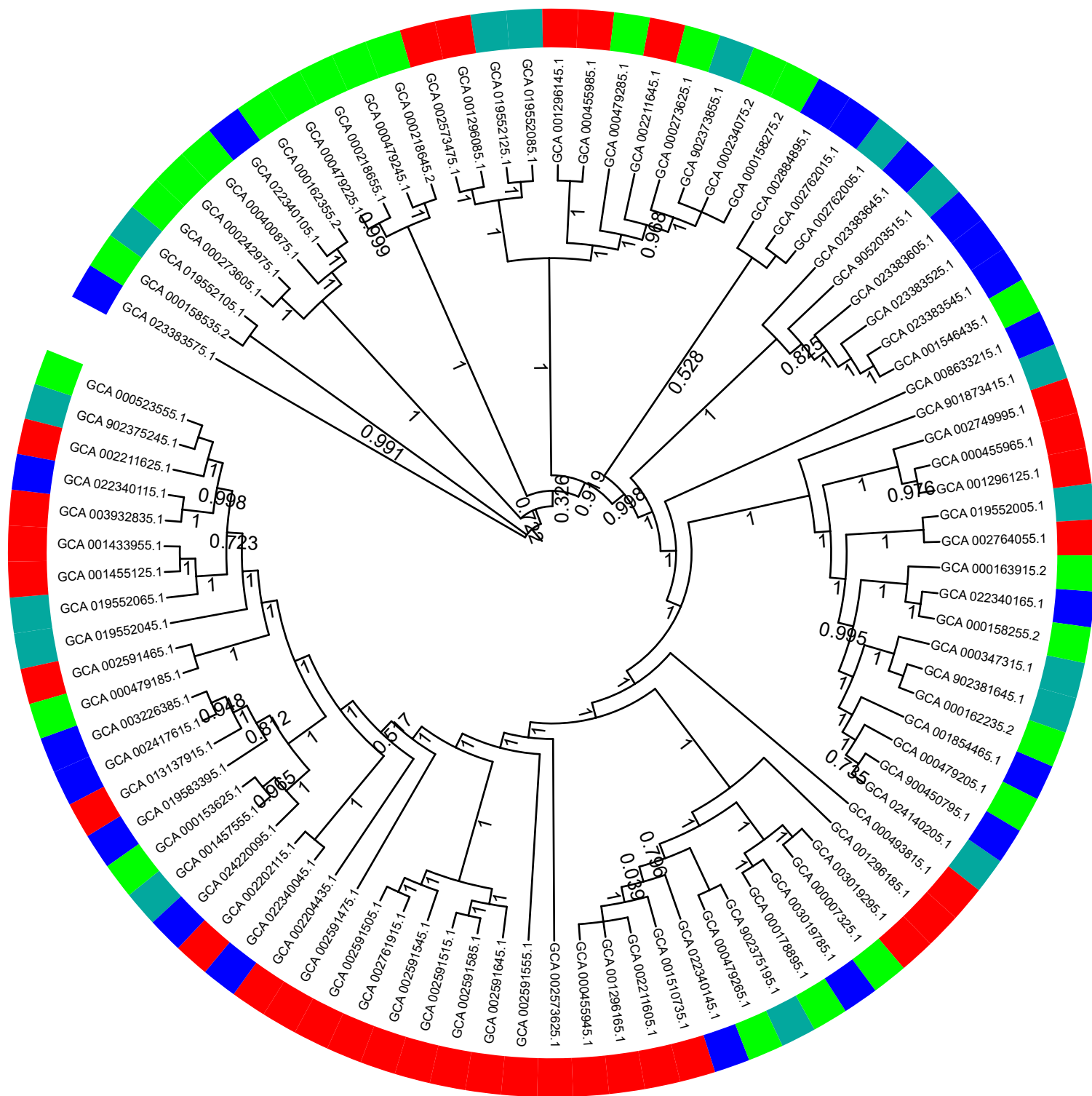

Supplement: Supplementary file 2 — Additional file 2: Figure S2. The phylogenetic tree of Fusobacterium nucleatum based on cgmlst markers. (Red represents mouth isolates, dark green represents gut isolates, blue represents other isolates and green represents unknown isolates.). [file 13099_2023_570_MOESM2_ESM.pdf]

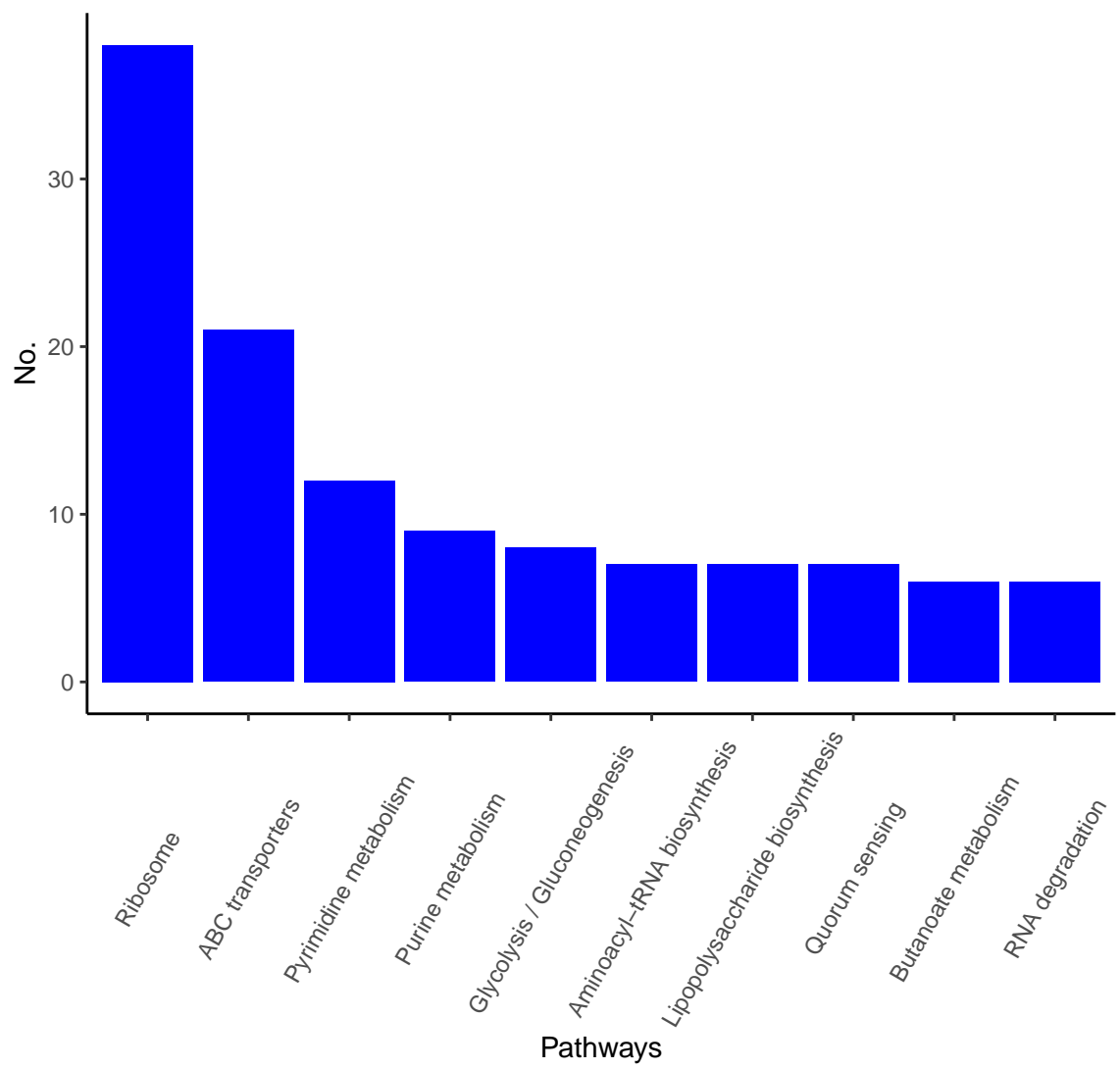

Supplement: Supplementary file 3 — Additional file 3: Figure S3. Functional enrichment of cgmlst marker genes. [file 13099_2023_570_MOESM3_ESM.pdf]

Gut

161

348

16

Mouth

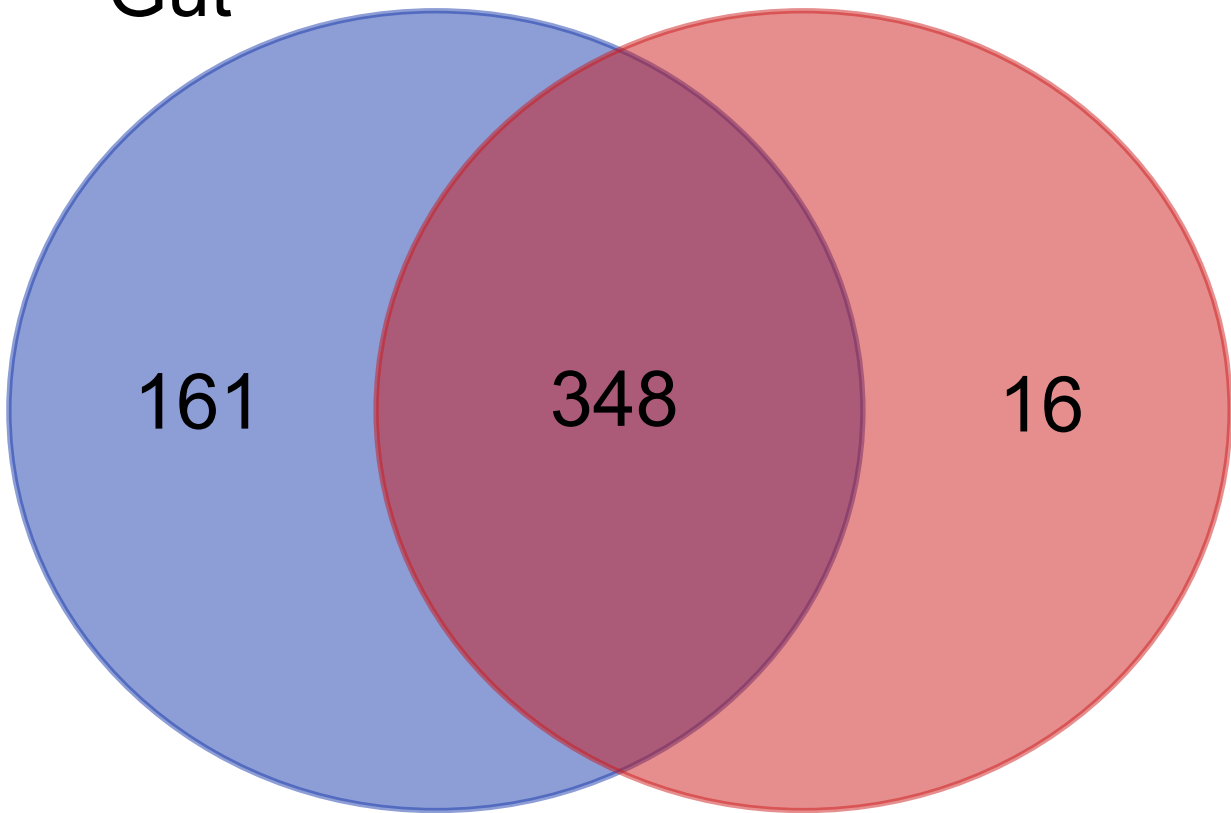

Supplement: Supplementary file 4 — Additional file 4: Figure S4. Venn diagram of cgmlst markers from mouth and gut isolates of Fusobacterium nucleatum. [file 13099_2023_570_MOESM4_ESM.pdf]
